# Child-street migration among HIV-affected families in Kenya: a mediation analysis from cross-sectional data

**DOI:** 10.1080/09540121.2016.1176672

**Published:** 2016-07-08

**Authors:** Michael L. Goodman, Miriam S. Mutambudzi, Stanley Gitari, Philip H. Keiser, Sarah E. Seidel

**Affiliations:** ^a^ University of Texas Medical Branch, Galveston, TX, USA; ^b^ Sodzo International, Houston, TX, USA; ^c^ Maua Methodist Hospital, Meru County, Kenya; ^d^ School of Public Health, University of Texas, Austin, TX, USA

**Keywords:** Street-involved children, street migration, social support, HIV, Kenya

## Abstract

Within Kenya, an estimated quarter of a million children live on the streets, and 1.8 million children are orphaned. In this study, we analyze how HIV contributes to the phenomenon of child-street migration. We interviewed a random community sample of caregiving women (*n* = 1974) in Meru County, Kenya, using a structured questionnaire in summer 2015. Items included reported HIV prevalence of respondent and her partner, social support, overall health, school enrollment of biologically related children and whether the respondent has a child currently living on the streets. Controlling for alcohol use, education, wealth, age and household size, we found a positive-graded association between the number of partners living with HIV and the probability that a child lives on the street. There was little difference in the odds of a child living on the street between maternally affected and paternally affected households. Lower maternal social support, overall health and school enrollment of biologically related children mediated 14% of the association between HIV-affected households and reporting child-street migration. Street-migration of children is strongly associated with household HIV, but the small percentage of mediated effect presents a greater need to focus on interactions between household and community factors in the context of HIV. Programs and policies responding to these findings will involve targeting parents and children in HIV-affected households, and coordinate care between clinical providers, social service providers and schools.

## Introduction

The HIV pandemic has been raging for the past three decades. Nowhere has the pandemic hit harder than Sub-Saharan Africa, and no population has been more affected than children (Sherr et al., 2014). The virus has left an estimated 15 million children orphaned (UNICEF, 2013); even when not orphaned, AIDS-affected children face staggering challenges (Cluver & Gardner, 2007; Mishra & Bignami-Van Assche, 2008; Richter & Desmond 2008; UNICEF, 2006). As the HIV pandemic matures into a chronic social problem, with fewer new infections and more people living longer with the illness, it is important to understand the full range of adversities posed to children in HIV-affected families. In this study, we explore whether children born into HIV-affected households are more likely to migrate to the streets than are other children. Further, we seek to identify potentially modifiable factors that may decrease risks posed to children living in HIV-affected households (Deeks, Lewin, & Havlir, 2013).

Millions of children live on the streets worldwide. In Kenya, there are an estimated 250,000 street-involved children and youth (SICY), a number that has likely increased over the past decade (Consortium for Street Children, 2002). SICY face many obstacles to flourishing, covering facets of physical, mental, social and cognitive health, as well as substance abuse, physical abuse and sexual abuse (Consortium for Street Children, 2002).

Research on push factors contributing to street-migration of children tends to rely on survey reports of children who are currently street-involved. Across Sub-Saharan Africa, SICY report leaving homes with inadequate food and parent-provided care and support (Plummer, Kudrati, & Yousif, 2007; Sorber et al., 2014). Globally, SICY report natal families with more children, parental alcohol use, parental mental illness and parental death than do non-SICY (Abdelgalil, Gurgel, Theobald, & Cuevas, 2004; McMorris, Tyler, Whitbeck, & Hoyt, 2002; Young, 2004). Where studied, SICY tend to not be enrolled in school, have completed fewer years of school than non-SICY and have biological siblings who are also not enrolled in school (Strobbe, Olivetti, & Jacobson, 2013; Young, 2004).

Given the deeply disruptive nature of HIV on child–parent dyads across Sub-Saharan Africa (Sherr et al., 2014), there is pressing need to understand the potential role HIV may play in street-migration. Prior research has shown children in HIV-affected households are more likely to experience abuse, neglect, parental death and poor health, parental alcohol use and school dropout (Cluver et al., 2013; Desmond et al., 2012; Fisher, Bang, & Kapiga, 2007). Additionally, HIV-infected mothers have higher gravidity than do non-HIV-infected mothers (Habib et al., 2008; Rollins et al., 2007), potentially increasing the risk of street-migration among children born into HIV-affected families.

### Study aim

We analyze the association between HIV-affected households and street-migration of children, and use multiple mediation analysis to explore hypothesized pathways potentially linking parental HIV with street-involved children. We hypothesized that social support, overall health, violent attitudes toward children, overall family functioning and school enrollment of biologically related children would carry significant portions of the effect of parental HIV on the street-migration of children, controlling for alcohol use, number of household children, maternal education, maternal age and household wealth.

## Methods

### Participants

Sample size for the study was determined based on financial and human resource limitations, as there was no known prevalence for households reporting a child lives on the street. Study subjects were selected using a stratified-random sampling approach. Twenty-three geographic clusters around Maua Methodist Hospital were selected due to ongoing hospital efforts in the area. Trained interviewers were assigned to neighborhoods in each cluster. A random-number-generated path was followed by each interviewer. Every other house was selected as a potential candidate for interview. Two inclusion criteria had to be met: (1) the household had at least one child currently living in the house and (2) the woman primarily responsible for caregiving duties was available to be interviewed. A total of 2129 were visited and found to have at least one child living at the home; of these, 51 women refused (2.4%) and 104 women were not at home (4.8%). A total of 1974 interviews were completed and included in this study. Interviewers did not return for more than one household visit.

### Survey instrument

The measures included in the present analysis included respondent-reported HIV status of herself and her partner, perceived social support, family functioning, subjective overall health, school enrollment of biological children living in the household, age, wealth index, years of completed schooling, number of children currently living in the household, respondent-alcohol use and whether the respondent had a child who currently lives on the street. The instrument was created in English, translated to Kimeru and then back-translated for comparison and refinement.

### Exposure measures

Self-reported HIV status was measured by asking the respondent whether she had ever been tested for HIV and whether the test returned positive. The respondent was asked the same pair of questions for her partner. In path analysis, the HIV-affected variable contained three levels – no reported HIV in the household, either respondent or her partner had a positive HIV test or both respondent and her partner received a positive HIV test. In regression analysis, there are four levels used – no reported HIV, maternal HIV only, paternal HIV only and dual affected are used as exposures.

### Outcome measure

Respondents were asked if all of their children were currently living at home, with living at home defined as “spending at least four nights a week for the past 6 months”. Respondents who replied that not all children were living at home were provided a list of other potential locations – with friends or relatives, in boarding school, on the streets or elsewhere. The outcome is a dichotomous variable – “yes” at least one child lives on the streets or “no” no child lives on the street. The “no” category includes children at other houses, away at boarding school or elsewhere.

### Mediator measures

Social support was measured using the multi-dimensional scale of perceived social support (MSPSS, Zimet, Dahlem, Zimet, & Farley, 1988; *α* = 0.94). The scale is a 12-item, 7-point Likert-type measure of emotional and practical support from 3 different sources – a special/romantic person, family and friends. The scale includes statements such as “there is a special person who is around when I am in need”. The original scale showed a three-factor structure, though in the present sample we found a strong single-factor solution.

Family functioning was measured using the general subscale of the McMaster Family Assessment Device (Epstein, Baldwin, & Bishop, 1983; *α* = 0.68). The measure evaluates perceived functioning of the family – including ability to coordinate, celebrate and support each other using 4-point Likert-type responses to items such as “we cannot talk to each other about the sadness we feel” (reverse coded).

Overall health was measured using the general self-rated health item “how would you describe your overall health at the moment” with four response options – excellent, good, fair and poor. General self-rated health is sensitive to a wide array of physiological and psychological maladies (Jylhä, 2009).

Respondents were asked how many biologically related children were living at home, again defined as “spending at least four nights a week for the past 6 months”. Respondents were also asked how many children under 18 years of age are currently enrolled in school. Households where all biologically related children are enrolled in school were compared to households where not all biologically related children are enrolled in school.

Violent attitudes were measured using the corporal punishment against children subscale of the Velicer Attitudes Toward Violence Scale (Anderson, Benjamin, Wood, & Bonacci, 2006). The subscale includes eight items measured with 5-point Likert-type response options (*α* = 0.72), using statements such as “children should be spanked for temper tantrums” and “giving mischievous children a quick slap is the best way to quickly end trouble”. We assumed a high probability of response bias among women reporting their child lives on the streets if responding to questions of past behavior, so employed an attitudinal scale instead.

### Control measures

Wealth was measured using a 9-item asset inventory, including electricity, radio, television, telephone, refrigerator, bicycle, motorcycle, car, land and added the number of rooms used for sleeping. The index showed a strong single-factor solution with a slight right skew.

Age was measured in years based on respondent self-report.

The number of children currently living in the house was used as a control measure due to its association with HIV, with both often being potential consequences of unprotected and more frequent sexual intercourse.

Years of completed schooling was also used as a control measure, and assessed the number of years of formal schooling the respondent reported as having successfully completed.

As problematic levels of alcohol consumption in natal families have been reported from street-involved children in the area, and alcohol is a determinant of HIV status (Fisher et al., 2007), we controlled for the behavior using a binary variable – any regular alcohol consumption vs. no reported alcohol consumption.

### Data analysis

**Figure 1.**
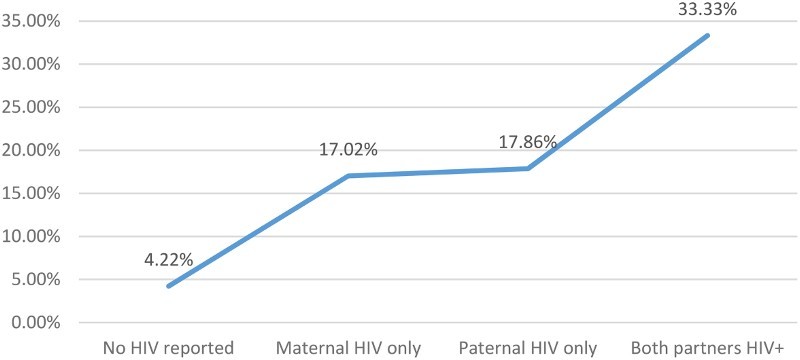
Percentage of respondents reporting a child lives on street by HIV status of parents.

**Figure 2.**
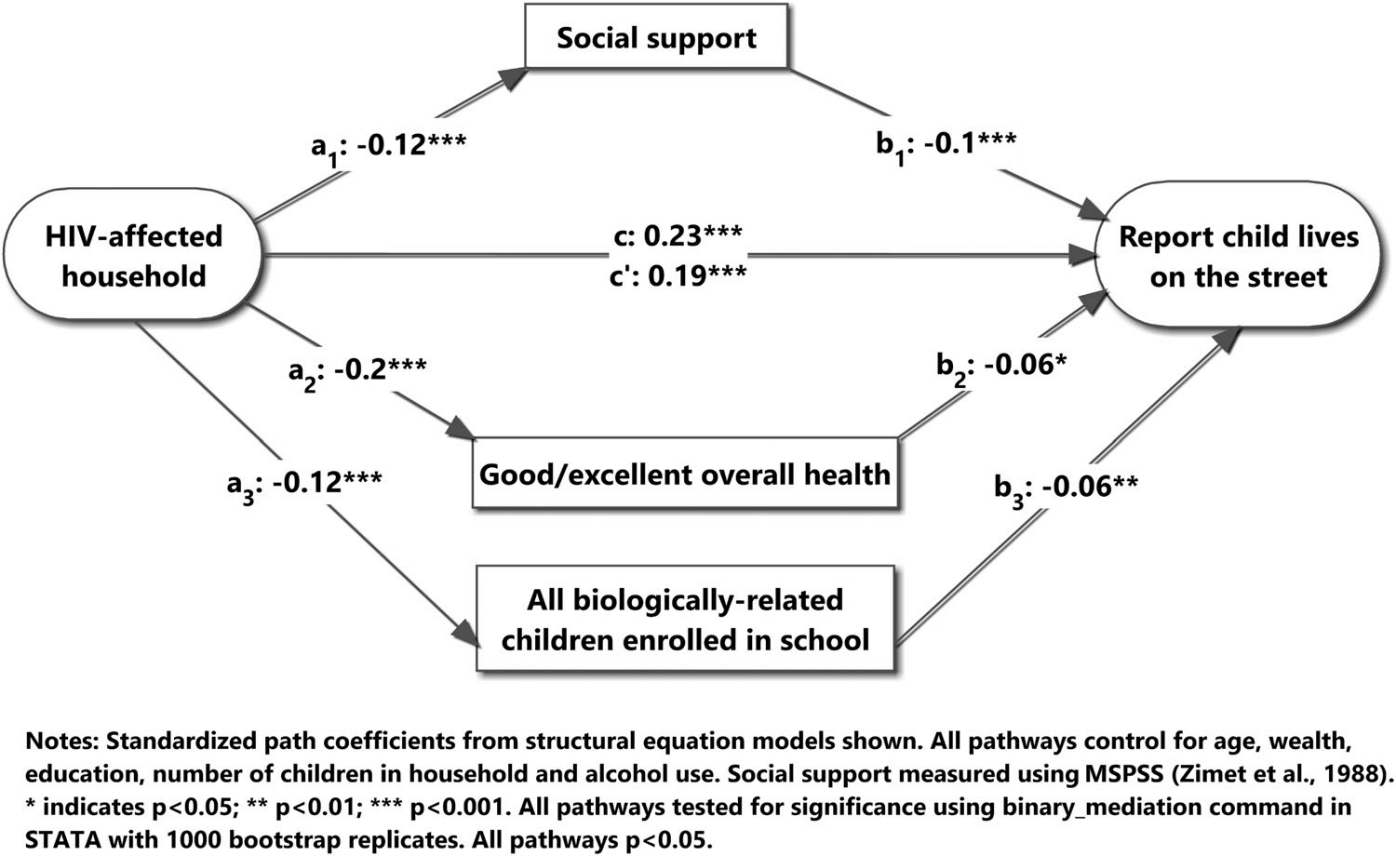
Path analysis of reporting a family child lives on the streets and HIV-affected households.

Bivariate descriptions of each control, outcome and mediator variable by HIV-affected and non-HIV-affected households are shown in Table 1 along with *t*-tests for continuous variables and Pearson *χ* ^2^ tests for categorical variables. The attributable fraction of households reporting child homelessness in the population is reported. Primary analysis used structural equation models to assess mediating pathways from living in an HIV-affected house to reporting that a child currently lives on the streets (Preacher & Hayes, 2008). Assessed mediators included: social support, family functioning, enrollment of biologically related children, violent attitudes toward children and overall health. Decomposition of effects (Table 2 was performed to assess and compare the percent of association carried by each mediator (Alwin & Hauser, 1975). The bootstrap approach (1000 reps) was used to determine the statistical significance (*α* = 0.05) of each pathway using the binary_mediation command in STATA v.13 (Ender, 2010). Figure 1 shows the percentage of households reporting a child lived on the street by HIV status of the household: no reported HIV, maternal HIV, paternal HIV and dual-affected HIV. Figure 2 shows the resulting conceptual model.

**Table 1.** Univariate and bivariate description of mediator and outcome measures, by reported HIV status.

	Univariate			No reported HIV			Respondent and/or partner reported HIV+			Test statistic (*p*)
	Mean (%)	95% CI		Mean (%)	95% CI		Mean (%)	95% CI		
Wealth index	4.55	4.43	4.68	4.69	4.56	4.81	3.8	3.48	4.13	t: 4.69 (<.001)
Age (years)	38.21	37.61	38.82	37.79	37.2	38.39	40.72	39.11	42.33	t: −3.24 (<.001)
Years of completed school	5.96	5.78	6.14	6.15	5.97	6.33	5.15	4.69	5.61	t: 6.15 (<.001)
Number of children in household	3.55	3.46	3.64	3.42	3.33	3.51	4.14	3.85	4.44	t: 3.68 (<0.001)
Self-reported alcohol use	7.54%	6.25%	8.84%	6.75%	5.54%	7.97%	17.24%	12.00%	22.48%	*χ*^2^: 27.31 (<.001)
Social support (7-point Likert-type scale)	5.42	5.36	5.48	5.5	5.44	5.56	4.87	4.72	5.03	t: 7.05 (<.001)
Overall health (4-point Likert-type scale)	1.85	1.8	1.9	1.97	1.93	2.02	1.15	1	1.3	t: 12.13 (<.001)
Family function (4-point Likert-type scale)	2.66	2.64	2.68	2.68	2.66	2.7	2.56	2.52	2.61	t: 3.67 (<.001)
Violent attitudes toward children (5-point Likert-type scale)	3.59	3.55	3.63	3.63	3.59	3.67	3.33	3.22	3.44	t: 4.96 (<.001)
All biologically related children enrolled in school	32.07%	29.69%	34.52%	33.55%	30.92%	36.25%	14.20%	9.22%	20.54%	*χ*^2^: 25.51 (<.001)
At least one family child lives on the street	6.49%	5.29%	7.69%	4.22%	3.15%	5.28%	22.35%	16.18%	28.51%	*χ*^2^: 98.79 (<.001)
Attributable fraction:							34.57%			
No HIV reported	88.52%	86.83%	90.06%							
Maternal HIV only	6.03%	4.90%	7.33%							
Paternal HIV only	1.80%	1.20%	2.59%							
Dual-affected HIV	3.66%	2.78%	4.71%							

Notes: Mediator, control and outcome variables stratified by reported HIV status. *T*-tests used for continuous and *χ* ^2^ used for categorical variables. Attributable fraction is the predicted percent reduction in households reporting at least one child lives on the street if HIV in the population were eliminated.

**Table 2.** Fixed-effects logistic regression of reporting at least one child currently lives on the streets.

	Child lives on the street					
	Model 1			Model 2		
	OR	95% CI		OR	95% CI	
No reported HIV	1	REF		1	REF	
Maternal HIV only	4.31***	2.14	8.68	3.65***	1.69	7.9
Paternal HIV only	4.12*	1.37	12.4	4.26**	1.32	13.77
Dual-affected HIV	5.33***	2.53	11.2	4.11***	1.81	9.33
Wealth index	0.9	0.8	1.02	1	0.88	1.13
Age (years)	1.01	0.99	1.03	1.01	0.99	1.03
Years of completed schooling	0.93	0.86	1.01	0.92	0.85	1
Household children (#)	1.18**	1.04	1.34	1.19*	1.04	1.36
Report alcohol use	1.58*	1	2.53	1.37	0.59	3.15
Social support (7-point scale)				0.8*	0.67	0.96
Good/excellent overall health				0.78*	0.61	1
Full school enrollment of biologically related children				0.5*	0.26	0.97
Wald *χ*^2^ (*p*)	77.2 (<.001)			82 (<.001)		

Note: Bootstrapped confidence intervals (1000 replicates) shown. *p < .05; **p < .01; ***p < .001. Social support taken from Zimet et al. (1988; *α* = 0.94). Model 1 shows HIV-affected household and control variables. Model 2 includes additional mediator variables.

Analogous regression models were calculated using fixed-effects logistic regression. Fixed effects were calculated for each geographic cluster from which observations were taken. The first models control for age, wealth, years of completed schooling, alcohol use and the number of children living in the household. The second models include the variables determined by bootstrap analysis to be significant mediators.

All data were recorded on paper, and entered into EpiData v.3. All analyses were conducted in STATA v.13.

### Ethical considerations

The study was approved by an ethics committee at Maua Methodist Hospital prior to data collection. Participants provided informed consent before being interviewed. The Institutional Review Board at the University of Texas Medical Branch provided ethical review and exemption before de-identified, secondary data were analyzed and presented for publication.

## Results

Mean respondent age was 38.2 (Table 1). The mean number of school years completed was 6, and the average household contained 3.6 children. Approximately 7.5% of respondents reported alcohol use. Approximately one-third of households reported full enrollment for biologically related children. Overall, 6.5% of households reported that a child lived on the streets. Approximately, 88.5% of households reported no HIV. Just over 6% of respondents reported maternal-only HIV. Just under 2% of respondents reported paternal-only HIV, and 3.6% of respondents reported dual-affected HIV. Women reporting they and/or their partners were living with HIV had significantly lower wealth, older age, fewer school years, more children in the household, lower social support, worse overall health, higher alcohol use, worse overall family function and higher percent of children living in the street. Women in HIV-affected households reported significantly less violent attitudes toward children.

Figure 1 shows the percentage of respondents reporting that a child lives on the street, by HIV status of the household. Respondents in households without any reported HIV had a 4.2% probability of reporting that a child lives on the street. The probability of reporting a child lives on the street was statistically equal between maternal HIV and paternal HIV households (17.02–17.86%). The probability of reporting a child lives on the street nearly doubles in dual-affected households (33.3%). The attributable fraction of households reporting a child lives on the street due to any reported HIV was 34.6% in the present sample.

Figure 2 shows the conceptual model of the path analysis. Controlling for wealth, age, education, reported alcohol consumption and the number of children in the household, HIV-affected households were significantly more likely to have a child currently living on the streets. The effect of parental HIV on child-street migration was mediated by lower social support (5%), worse overall health (5%) and lower probability of having all biologically related children currently enrolled in school (3.6%). Family functioning and violent attitudes toward children were not found to be significant mediators. In total, 86% of the pathway from parental HIV to child-street migration was unmediated by observed mediators.

Table 2 shows two fixed-effects logistic regression models. The first shows that, before accounting for mediators, the odds of reporting that a child lives on the street increases in a linear fashion for each level of HIV-affected household, with nearly equivalent odds for maternal (OR: 4.31, 95% CI: 2.14–8.68) and paternal HIV (OR: 4.12, 95% CI: 1.37–12.4). Dual-affected households had odds of a child living on the street that were 5.3 times those of households without reported HIV. Of the control variables, the odds of a child living on the street increased 18% for each additional child in the house, and 58% among women who reported regular alcohol consumption.

After including the mediating variables, we found that a one-unit increase in the average-item response on the social support scale predicted a 20% reduction in the odds of a child living on the street. Respondents who were in excellent or good self-rated health had 22% lower odds of reporting a child lives on the streets. Respondents who reported that all biologically related children under 18 years of age were enrolled in school had 50% lower odds of reporting a child currently lives on the street. As seen in Figure 2 and Table 3, social support, overall health and school enrollment of biologically related children significantly mediate the pathway of parental HIV and child-street migration. Comparing the odds ratios in Model 1 and Model 2, the most sizable reduction in magnitude after including the mediators occurs among dual-affected households followed by maternal-only HIV. There is no reduction in odds ratio for paternal-only HIV after including the mediators.

**Table 3.** Decomposition of direct, indirect and total effects of HIV-affected household and street-migration of children.

Path notation	HIV-affected -> Child lives on the streets				% Total effect
C				0.23	
c′	Direct Effect			0.19	85.59
a_1_ * b_1_	Social support	−0.12	−0.1	0.012	5.41
a_2_ * b_2_	Good/excellent overall health	−0.2	−0.06	0.012	5.41
a_3_ * b_3_	Full school enrollment of biologically related children	−0.12	−0.07	0.008	3.78
Total indirect effect				0.032	14.59
Total effect				0.222	

Notes: Decomposed effects use standardized, survey-adjusted coefficients from structural equation modeling. Bootstrapped confidence intervals (1000 reps) were calculated for each pathway to establish statistical significance. Each pathway is significant at *p* < .05. Conceptual model found in Figure 2.

## Discussion

In a world of chronic HIV, where children remain most vulnerable due to their dependency on caregivers who may be unable to provide adequate care, identifying new areas of risk for children and potential methods to mitigate these risks are imperative for child well-being. We found that the odds of a mother (or other caregiving woman) reporting that her child lives on the streets were over five times higher if both she and her partner are living with HIV. These odds decreased slightly when considering social support, overall health and school enrollment of other biologically related children, which mediated 14% of the association.

The odds of reporting a child lives on the street were surprisingly equivalent for maternal HIV only and paternal HIV only, suggesting either parent living with HIV should be targeted by family strengthening programs to reduce child-street migration. Regression models showed change in the odds ratios of maternal HIV only and dual-affected, but not paternal HIV only, after including mediating variables. This suggests that the pathway between paternal HIV and child-street migration is not mediated by maternal social support, overall health or school enrollment of biologically related children. Further research into mechanisms by which parental HIV leads to vulnerabilities for children, including street-migration, should consider potential gendered effects. As over one-third of the risk for reporting a child lives on the streets is due to HIV (attributable risk fraction = 34.6%), further research into other mechanisms by which parental HIV may place children at risk of living on the streets is required to articulate relevant policy and program interventions.

There are numerous potential mediators explaining why children of parents living with HIV are at greater risk of living on the streets, including unobserved psychosocial, educational and financial constraints. AIDS-related stigma has explained both experiences of bullying and school dropout in the Sub-Saharan African context (Cluver et al., 2013). While evidence is less available for pre-street experiences of bullying, school dropout is a risk factor for living on the streets among children in East Africa (Henley, McAlpine, Mueller & Vetter, 2010). While it is parents who are infected by HIV, community-wide responses are required to prevent risk of youth migration to the streets in HIV-affected households. Henley et al. (2010) found that promoting school attendance could reduce the natal family-street migration, presenting a potential mechanism to mitigate one pathway found in these data. Campbell et al. (in press) suggest caution against overly ambitious policy plans to use schools in Sub-Saharan Africa to strengthen family challenges due to strains on teachers.

Conceivably community-based organizations can partner with schools to target and intervene with children at risk of dropping out, which may reduce the risk of children from AIDS-affected families migrating to the streets. Community-based organizations already provide a number of service benefits, though these are largely unexplored (Yakubovich et al., 2016). Research into policy and funding environments conducive to effective community-based organizations, and their partnership functions with schools, may illuminate a meaningful path forward to AIDS-resilient communities where all children can flourish. Given the wide range of sectors required to protect children in HIV-affected households – here including social support, school enrollment and health systems – policy support for community-based organizations may enable more relevant, timely and coordinated support for HIV-affected families. Such organizations could, for example, help coordinate early childhood education for children in HIV-affected homes with other social and health services targeting parents living with HIV. Early childhood education has been consistently found to encourage school completion and promote success across the life span (Irwin, Siddiqi, & Hertzman, 2007), and may help reduce street-migration of children in HIV-affected families.

There are several limitations to our study. We used overall health as a summative, sensitive measure of the respondent’s health. Biomedical, financial and psychosocial factors may influence the overall health of women living with HIV (Brandt, 2009; Phaladze et al., 2005), and a general health measure does not help policy-makers decide where to place resources. We did not measure length of time since diagnosis, age of child on the street or other biologically meaningful markers of HIV – viral load, CD4 count, other opportunistic infections, etc. These indubitably would have some effect on the welfare of children.

Data used for the study were based on self-report which is vulnerable to recall, misinterpretation, simplification and social desirability bias (Zandwijk et al., 2015). Further, there are culturally bound conceptions of social support and overall health such that translation and inference in English language about meanings held in Kimeru culture are bound to not completely align with local understandings (Kim, Sherman & Taylor, 2008).

Despite these limitations, we believe that this study makes novel contributions to understanding some ways in which adult HIV impacts the welfare of children. To our knowledge, it is the first study in Sub-Saharan Africa to assess the mediating effects of maternal social support, overall health and school enrollment on child homelessness among HIV-affected households.

## Conclusion

Using empirical data, we identify higher odds that mothers report their children have migrated to the streets among HIV-affected households. Only a small percentage (14%) of the association was explained by observed mediators – lower social support, lower school enrollment and worse overall health. Areas for future exploration to define pathways between HIV-affected households and street-migration of children include disease progression, social stigma and risk of school dropout. Additionally, qualitative research with HIV-affected households may help interpret these findings. The policy and funding environments should consider mechanisms of empowering local community organizations to coordinate responses to novel and cross-sectoral challenges facing HIV-affected families.

## Disclosure statement

No potential conflict of interest was reported by the authors.
